# Disclosure of human immunodeficiency virus status to children: Pattern followed by parents and caregivers

**DOI:** 10.4102/phcfm.v12i1.2230

**Published:** 2020-11-23

**Authors:** Cebsile P. Dlamini, Mokgadi C. Matlakala

**Affiliations:** 1 Department of Health Studies, College of Human Sciences, University of South Africa, Pretoria, South Africa

**Keywords:** caregiver, disclosure, HIV, status, parent, pattern

## Abstract

**Background:**

Disclosure of human immunodeficiency virus (HIV) status may be perceived as simply the process of revealing a person’s HIV status, whether positive or negative. Despite the emerging evidence of the benefits of disclosure, who, when and what to disclose to a HIV-infected child remains a challenge.

**Aim:**

This article reports on the patterns of HIV status disclosure to the infected children by their parents and caregivers.

**Setting:**

The study was conducted in the outpatient clinic of one referral hospital offering comprehensive HIV care in the Lubombo region, eSwatini.

**Methods:**

A qualitative descriptive design was followed. Data were collected through semi-structured individual interviews with a purposive sample of 13 parents and caregivers whose children were on antiretroviral treatment and collecting treatment from the specific outpatient clinic. Audio recorded data were transcribed verbatim, thematic content analysis was done and used to organise and present the findings.

**Results:**

Four themes that emerged in relation to the topic of patterns of disclosure were disclosure of HIV status as a process rather than an event, a proposed person to disclose the HIV status to the child, the appropriate age to disclose HIV status to a child and type and amount of information to give in relation to the HIV status. The proposed person to disclose the HIV status to the infected child was the parent or caregiver involved as the primary carer of the child. There was no agreeable appropriate age to disclose HIV status to an infected child and the type and amount of information to disclose varied with the individuals depending on what prompted disclosure.

**Conclusion:**

Human immunodeficiency virus disclosure to children demands parents’ and caregivers’ participation and their knowledge of child development.

## Introduction

Human immunodeficiency virus (HIV) affects both adults and children because of its mode of transmission as in mother-to-child transmission of HIV. The use of antiretroviral therapy (ART) has become widely used for HIV and is available in developing countries including eSwatini. This increases the number of HIV-infected children surviving into adolescence and beyond. Therefore, as the children grow older, they need to know the facts about the disease and the treatment they are taking. Without knowing their HIV status whilst infected, adolescents may start engaging in sexual practises and infect others unknowingly, thus spreading the disease.

In eSwatini, 13 000 children aged 0–14 years were living with HIV in 2017 of whom 75% were on antiretroviral treatment.^1,2,3^ Antiretroviral therapy has been free in eSwatini since 2003.^4^ In 2014, eSwatini adopted the 2013 World Health Organization (WHO) guidelines of which one is that anyone diagnosed with HIV should be started on ART regardless of their CD4 count. This meant thousands more people including children were eligible for the treatment.^5,6,7^ However, it is not clear if the children were told about their HIV status and ART. The adopted guidelines on the use of antiretroviral drugs for treating and preventing HIV infection indicate HIV testing and counselling (HCT) as the first step to treatment and prevention.^8,9^ The guidelines further suggest that all children receive early HIV diagnosis and get linked immediately to appropriate care. Human immunodeficiency virus testing and counselling policy recommends disclosure of HIV status between parents and children.^10^

Disclosure is vital in the management and care of HIV.^11^ Human immunodeficiency virus status is usually disclosed voluntarily by the index person, but it can also be revealed by others with or without the other person’s consent.^12^ It is not only important for children but even adults are encouraged to disclose their HIV status to significant others, especially their families and partners. The eSwatini Ministry of Health^13^ integrated HIV guidelines indicate that prior to initiation of ART the client should bring a treatment supporter to the clinic. This treatment supporter should be someone trusted by the client and should be aware of the HIV status of the concerned client.^13^ In the clinic where this study was conducted, the children on ART were always accompanied by their parents or caregivers. However, the observation of the researcher was that during treatment refills, some of the children did not seem to know their HIV status, even though they were already on ART. Therefore, it was of interest for the researcher to investigate the pattern of disclosure of HIV status to children.

Human immunodeficiency virus disclosure may be perceived as simply the process of revealing a person’s HIV status, whether positive or negative.^14^ The disclosure poses challenges to the healthcare providers and the parents or caregivers of HIV-infected children.^15^ It is indicated that generally the challenges of HIV disclosure remain stigma and discrimination, including in eSwatini,^16^ behaviours associated with transmission routes, feelings of shame and fear of unintentional disclosure of family illness.^17^ Furthermore, who, when and what to disclose to the infected child is still a huge challenge to the parents and caregivers. It is therefore necessary to understand the complexity of HIV status disclosure to HIV-infected children by describing the pattern of disclosure used. In the case of children who are HIV infected, we believe that disclosure decisions lie with the parent or caregiver in collaboration with the healthcare providers.

Human immunodeficiency virus disclosure entails communication about a potentially life-threatening, stigmatised and transmissible illness. Disclosure to children is beneficial to their self-esteem, develop a sense of autonomy and empowerment and help them to participate in their own medical treatment.^18^ In eSwatini, there are no support guidelines that are in the collective context of Swazis on HIV status disclosure to infected children by their parents or caregivers. Some parents lack skill on disclosure as to when, how and what information to give to the infected child.

## Aim

The aim of this study was to describe the pattern of HIV status disclosure to the infected children by their parents and caregivers.

## Research methods and design

A qualitative descriptive design was used and it provided an opportunity for the researchers to have an in-depth and holistic understanding of patterns of disclosure of HIV status to the infected children, by collecting rich narrative data from the parents and caregivers.^19,20^

### Setting

The study was conducted at the outpatient clinic of a major referral hospital offering comprehensive HIV care in the Lubombo region, eSwatini. The hospital is servicing over 10 clinics in the region and both children and adults do their consultation at the hospital. This setting was deemed the most appropriate for this study as the participants were interviewed during theirs and their children’s monthly follow-up appointments for ART.

### Study population and sampling strategy

The population for this study comprised of parents and caregivers of HIV-infected children aged between 6 and 15 years. Non-probability purposive sampling was used to select the participants who had disclosed or not disclosed the status to their children. To be included in the study, the participants had to be parents or caregivers of HIV-infected children, who do the monthly refills of ART for the children or themselves at the specific outpatient clinic of the selected hospital. The participants were identified and approached by the researcher during their regular clinic visit and the study was explained by the researcher upon contact. Recruitment was done on an individual basis and for those who agreed to participate in the study, a consent form was signed following a thorough explanation of the aim of the study and the expectation of the participants in taking part in the interviews.

### Data collection

Semi-structured individual interviews were used to collect data.^19,20^ After permission was granted, the researcher got the names of the eligible participants through the assistance of the healthcare workers in the HIV clinic, whereby the participants were contacted for recruitment. The goals of the study were stated and how the data would be used. A purposive sample of participants who met the inclusion criteria participated in the interviews. They were interviewed on an individual basis in a private room at the clinic. The interviews took approximately 30 min each and were audio-recorded with the permission of the participants. The semi-structured individual interviews were conducted until data saturation was reached with the 13th participant.

### Data analysis

Audio-recorded interviews were transcribed verbatim. Data analysis was done manually by two researchers using open and focused coding^21^ to identify prominent themes and descriptive and meaningful labels to data patterns amongst themes.^19^ To ensure rigour and trustworthiness, validation of the information of the interviews were done from the participants at the end of each interview, and a consensus meeting was held by the researchers to have an agreement on the main themes of the results.

### Ethical consideration

Ethical clearance was sought and obtained from the Health Studies Higher Degrees Committee, Unisa (HSHDC/434/2015). Permission to conduct the study was obtained from the hospital in writing (MH/5999C/FWA 000 15267/IRB 000 9688). The participants were informed about the type of data to be collected and the procedure to be followed. They were also informed about their selection from the appropriate population. Participants were assured that there would be no physical risk in this study but that emotions may be triggered and psychological trauma would be cared for by a dedicated psychologist. Participants were assured that confidentiality would be maintained with all data and complete anonymity was guaranteed. An informed consent was obtained from the participants and verbal consent to be audio-recorded was obtained following a thorough explanation of the purpose of audio recording. Their privacy was respected throughout. Participation was strictly voluntary, and participants had the right to withdraw from the study at anytime or to refuse to provide any particular information.

## Results

The participants were 2 males and 11 females, aged 26–70 years and related to the children infected with HIV as parents or caregivers. Only two participants had not disclosed the HIV status to their children but had done so to significant others.

The study revealed that parents and caregivers were faced with the difficult decision of HIV status disclosure to their infected children. Disclosure occurred in various patterns. The prominent themes that influenced the pattern of disclosure of HIV status to the infected children are as indicated in Table 1.

**TABLE 1:** Themes reported to influence disclosure of human immunodeficiency virus status to children.

Topic	Themes
The pattern followed on disclosure of HIV status to the infected children	Disclosure of HIV status as a process rather than an event Person to disclose the HIV status to the child The appropriate age to disclose HIV status to a child Type and amount of information to give in relation to the HIV status

HIV, Human immunodeficiency virus.

The themes presented in Table 1 are described here, with supporting quotes from participants.

### Disclosure of human immunodeficiency virus status as a process rather than an event

The participants had a general understanding of the concept of disclosure and indicated that an attempt should be made to disclose and assist children to understand their HIV status. Disclosure was viewed as a process that should be ongoing as the child grows from childhood to adolescence and adulthood. However, they lacked consensus or even knowledge and guidance on the steps to follow in the process. The general view was that children should be told about their HIV status whilst they are still young, although some of the participants had not told their children.

The participants mentioned that when the child is still young she or he may not understand the concept of HIV or even disclosure, and therefore it may not be beneficial to give detailed information on the disease. They were of a view that the child should be told in stages and according to the age; as disclosure is meant to be an ongoing process. Thus, their reason for disclosing to significant others, other than the child. The following were mentioned:

‘I think it is important for the child to know in a simple manner. A child may be confused as to what are you talking about being HIV positive.’ (Participant 6, female caregiver to 8-year-old girl)

‘I can say that this should be done in stages and see the child’s reaction.’ (Participant 7, female parent to 15-year-old boy)

‘I will keep on telling her as she grows because she cannot understand much about HIV now.’ (Participant 12, female parent to 7.5-year-old girl)

‘He should know his HIV status as he grows so that he does not stop taking the medication.’ (Participant # 2, male caregiver to 7-year-old boy)

Some participants had disclosed their personal HIV status and the status of the child to significant others, such as family members or trusted people in the community like school teachers, other than disclosing it to the infected child. Those who did, had not fully disclosed the HIV status to the infected child. This signified clearly that disclosure is important in HIV management and care.

‘My condition is not a secret to the family and community members. I also disclosed to all of them about the status of the child. Even at school the head teacher and other teachers are aware that this child is on HIV medication. But the child does not know yet.’ (Participant 7, female parent to 15-year-old boy)

‘My family knows especially my mother and my siblings. Some community members know about it and I am the one who told them. I will tell the child as she grows.’ (Participant 10, female parent to 6-year-old girl)

### Person to disclose the human immunodeficiency virus status to the child

This theme emerged in relation to who must disclose the status to the child. The parents and caregivers, including those who had not disclosed the HIV status to their children indicated that they preferred to personally tell the children about their HIV positive status, as indicated in the following:

‘It is important that we tell our children about their status and be truthful how they got infected. As they grow they will understand how HIV can be transmitted.’ (Participant 8, female parent to 8-year-old boy)

‘I think I can be brave to tell her myself.’ (Participant 5, female caregiver to 11 year old girl)

‘I just felt I should do it on my own [*referring to disclosure of the child HIV status*] because I wanted her to understand why she has to take the medication.’ (Participant 3, male parent to 9-year-old girl)

Interestingly, when asked who else would be appropriate to disclose or help to disclose the HIV status to the child, the need for a counselor to tell the child about his or her HIV status was indicated by one participant as gathered from this statement:

‘I think the counselor can help me since I am afraid on my own.’ (Participant 1, female parent to 7-year-old boy)

### The appropriate age to disclose human immunodeficiency virus status to a child

The issue of when to disclose had variations based on different reasons perceived by the participants on when to disclose or not to disclose the HIV status to the infected child and situations encountered by each parent or caregiver. There was no definite age agreed upon, but some disclosed to their children aged between 6 and 10 years. The cognitive level of the child was a factor that indicated to some of the participants who were of an opinion that the children did not have a good understanding of HIV when young. This was in relation to the children seldom questioning the medication that they were taking. A revelation as expressed by the participants was that when the children questioned the medication the parent or caregiver had to disclose prematurely, that is, before the proposed age of readiness for disclosure. Therefore, besides the appropriate age, disclosure was done sometimes when questions were asked. The following quotes explain:

‘It is important that the child should know about her status, but it is a difficult task. I also told her last year at the age of 6 years.’ (Participant 11, female parent to 7-year-old girl)

‘When she was around 6 years old, she asked about the medication and my mother told her that she should take her medication.’ (Participant 6, female caregiver to 8-year-old girl)

‘I can say that it is important to tell the child at least around the age of 10 years.’ (Participant 7, female parent to 15-year-old boy)

‘This child is clever sister; he was asking more questions about the medication he was taking every day. He even asked why he was the only one among the children who was taking the medication.’ (Participant 9, female caregiver to 9-year-old boy)

### Type and amount of information to give in relation to the human immunodeficiency virus status

This theme related to what to tell or disclose to the child. There were different views on the information to be imparted from parents to children who are HIV positive and whether to tell their children about their HIV status. Although there was a consensus amongst the participants on the importance of disclosure, the type of information they disclosed varied. Some had not given any information about HIV or the medication to their children, until the child asked. The parents and caregivers were rather evasive or misleading about information to tell instead of full disclosure of HIV status. The following were mentioned:

‘I have told her that she is sick and should take her medication for the rest of her life, but I did not openly tell her that she has HIV.’ (Participant 12, female parent to 7.5-year old-girl)

‘I have not openly told him that he is HIV positive; but just told him that the medication he is taking is important that he takes it every day of his life. I even told him that I am also taking the same medication.’ (Participant 8, female parent to 8-year-old boy)

On the other hand, some participants had not given any information or gave only partial information without actually naming the disease, whilst others gave different reasons for the questions asked by the children:

‘No, I have not openly told her that she has HIV, but one nurse told her that she was taking the medication because she is sick. She does not know about the sickness.’ (Participant 10, female parent to 6-year-old girl)

‘I only told her that her blood is not clean, so that is why she should take the medication.’ (Participant 5, female caregiver to 11-year-old girl)

## Discussion

Parents and caregivers lacked consensus on the appropriate pattern of disclosure of HIV status to their infected children. Understanding disclosure of HIV status as a process rather than an event concur with the guidelines^9^ on HIV disclosure counselling for children up to 12 years of age, which state that disclosure leads to extended discussions as the implications of having HIV in the family unfold. The guidelines further state that, such discussions do not occur as a tactful, one-time event, but they occur over time.^9^ Literature showed that HIV disclosure is a process that moves a child from a state of nondisclosure to partial, and then full disclosure of illness.^22^

The parents and caregivers in this study disclosed the HIV status of their children at the concrete operational stage, which is between 6 and 10 years of age. Piaget’s theory of development^23^ characterised the concrete operational stage by concrete-logical reasoning, where children are able to make multiple connections of causation to a single phenomenon and can understand hierarchies.^23^ Children are able to identify an external cause as the source of their illness, identifying it as something undesirable. At this stage, children are in a better position to understand illness.

The findings of this study were slightly different from Piaget’s theory of development as some parents had partially disclosed the HIV status to their children at the age of 6 years. According to Piaget’s theory the formal operational stage is from age 11 years and above. Piaget characterised this stage by formal-logical thinking, where children begin to think hypothetically and abstractly when they can correlate multiple causations to illness, including external and internal factors related to their health.^23^

With regard to when to disclose, theappropriate age to disclose HIV status to a childrelate with the paediatric HIV disclosure process-oriented framework, which encourages disclosure of HIV-infection status to school-age children and recommends that adolescents should know their HIV status. Children generally start primary school at the age of six (6).^22,24^ Parents of adolescents in this study had disclosed their status to them partially.

With regard to what to tell the child, the parents and caregivers did not give full disclosure to their children. Full disclosure is when the child is provided explicitly with the name of the illness he or she is suffering from. The pathophysiology, mode of transmission and the importance of taking medication are fully explained. In addition, all questions asked are truthfully answered and support is given.^23,25^ The partial disclosure of the participants’ children’s HIV status could be related to the age of the child.

The findings on the type and amount of information to give in relation to the HIV status, revealed some aspects of dishonesty. Dishonesty of HIV status related to ascribing the child’s condition to a different illness or linking the child’s medication and some dissimilar physiology (e.g., linking ART to not having clean blood) frequently coupled with non-disclosure. The results concur with findings on patterns of disclosure of HIV status to the infected children in sub-Saharan Africa,^25,26,27,28^ which indicated that there are various patterns of disclosure: ranging from no information provided, deflecting information provided, partial information provided to full information provided.

The findings in this study indicated that disclosure occurred in various patterns. This refers to the procedure or way in which information was imparted from parents or caregivers to the children who are HIV positive with respect to who, when and what to disclose. The information contributes to an understanding of HIV disclosure patterns and a greater insight into the therapeutic relationship of parents and caregivers with their HIV-infected children.

## Limitations of the study

There is still a stigma attached to HIV, and generally some people prefer to say less about it even if they have fully accepted their status. The participants might have withheld the information as it might expose their ignorance or lack of knowledge regarding the appropriate information to disclose to their infected children. The study was conducted in one hospital in the Lubombo region of Swaziland, which has four regions, and therefore the findings could not be generalised to all the hospitals and communities for eSwatini. Only caregivers and parents were included in the study. Therefore, including children who are HIV positive could provide a bigger sample and more comprehensive results on who, when and what to disclose to HIV positive children.

## Conclusion and recommendations

Human immunodeficiency virus disclosure in children can be used to improve health outcomes.^29^ However, disclosure demands parents’ and caregivers’ participation, knowledge of child development, insight into the emotional, cognitive and social well-being of the child depending on various circumstances. The reality was the difficulty of disclosure when one does not understand when and what to disclose. The findings can be used to design context-specific measures in HIV prevention, treatment, care and support services.

Disclosure depends on various circumstances. Therefore, it is recommended that there be a standard pattern and guidance on disclosure regarding to whom and when to disclose the HIV status to the infected children. In addition, there should be a standard disclosure plan in health facilities indicating the amount and type of information (what) that will be shared with parents and caregivers to enable them to disclose age appropriate information to the infected children. It is important to allow parents and caregivers to verbalise their concerns and challenges on disclosure of HIV status to the infected children.
